# The association of three DNA repair genes polymorphisms on the frequency of chromosomal alterations detected by fluorescence in situ hybridization

**DOI:** 10.1007/s00420-021-01652-8

**Published:** 2021-03-28

**Authors:** Fábio Santiago, Rafaele Tavares Silvestre, Ubirani Barros Otero, Marianne Medeiros Tabalipa, Marilza de Moura Ribeiro-Carvalho, Luciano Rios Scherrer, Ahmed Al-Rikabi, Thomas Liehr, Gilda Alves, Maria Helena Ornellas

**Affiliations:** 1 Laboratory of Circulating Biomarkers, Department of Pathology, Faculty of Medical Sciences, Rio de Janeiro State University (UERJ), Avenida Professor Manuel de Abreu 444, 4° andar, Vila Isabel, Rio de Janeiro, 20551-030 Brazil; 2 Graduation Program of Medical Sciences (PGCM), Medical Sciences Faculty (FCM), Rio de Janeiro State University, Rio de Janeiro, Brazil; 3 Technical Area Environmental, Work and Cancer, Coordination of Prevention and Surveillance, National Cancer Institute José Alencar Gomes da Silva (INCA), Rio de Janeiro, Brazil; 4 Kennedy Faculties of Belo Horizonte, Minas Gerais, Brazil; 5 Jena University Hospital, Friedrich Schiller University, Institute of Human Genetics, Jena, Germany

**Keywords:** Benzene, Gas station worker, *RAD51*/*G135C*, *ATM*/*P1054R*, Chromosome aberration

## Abstract

**Purpose:**

Gas station workers (GSWs) are exposed to carcinogenic agents. The aim was to study the association of high somatic chromosome alterations (CAs) rates in the blood of GSWs and the polymorphisms of three genes playing a role in DNA double-strand break repair.

**Methods:**

This is a cross-sectional study with 114 GSWs and 115 age-matched controls. Cytogenetic analyses, blood exams, medical interviews and genotypes for *RAD51*/*G135C* (rs1801320), *ATM*/*P1054R* (rs1800057) and *CHEK2*/*T470C* (rs17879961) genes were performed.

**Results:**

The CA rate in GSWs was 9.8 CAs/1000 metaphases, and 19.1% of the workers had > 10 CAs per 1000 metaphases (group two). GSWs had decreased levels of monocytes (*P* = 0.024) in their blood exams. The number of variant alleles of the *RAD51*/*G135C* polymorphism was higher in GSWs (*P* = 0.011) compared to the controls, and were associated with enhanced number of CAs per worker (*P* = 0.008). No allele variant was found for *CHEK2*/*T470C* in this study.

**Conclusion:**

The *RAD51*/*G135C* polymorphism appears to be related to genome instability in gas station workers. Increasing the knowledge of DNA repair gene variations involved in maintaining genomic stability in GSWs may be crucial for future cancer prevention.

## Introduction

BTEX (benzene, toluene, ethyl benzene, and xylene) are aromatic hydrocarbons widely used as solvents in fuels, being chemical contaminants in gas stations´ environments. Benzene is considered the main carcinogenic agent (group one according to IARC), and the association with cancer is well-established (IARC 2018; Falzone et al. 2016). In Brazil, as self-service fuel filling at gas stations is illegal, gas station workers (GSWs) have to fill the fuel in the car’s tank; due to this, they are chronically exposed to high concentrations of BTEX. Although the mechanisms by which BTEX cause genotoxic effects are not fully clear, there is evidence that the function of a wide range of cellular targets are perturbed by specific metabolites and reactive oxygen species (ROS). Genotoxic effects include: (one) inhibition of topoisomerase II; (two) adduct formation of reactive metabolites; (three) oxidative DNA damage; (four) error-prone DNA repair; and (five) epigenetic alterations (McHale et al. 2011; Dewi et al. 2020).

Chromosome alterations (CAs) are standardized biomarkers of early biological effects in human biomonitoring. In fact, CAs in peripheral blood lymphocytes reflect inter-individual sensitivity to exogenous genotoxic substances and can be used as biomarker of carcinogenic risk (Rossner et al. 2005; Mateuca et al. 2012; Zhang et al. 2012; Li et al. 2015; Villalba-Campos et al. 2016). Fluorescence in situ hybridization (FISH) using whole chromosome painting (wcp) probes allows a rapid detection of CAs, enabling new possibilities of cytogenetic dosimetry (Verdorfer et al. 2001; Santiago et al. 2014).

Lower activity of the DNA repair mechanisms may generate higher somatic rates of CAs, favoring the development of cancer (De Palma and Manno 2014). The DNA repair genes *RAD51*, *ATM*, and *CHEK* play a role in the DNA double-strand break repair preventing CAs; however, some polymorphisms could made this task less efficient.

The aims of this study were to assess the frequency of the *RAD51*/*G135C* (rs1801320), *ATM*/*P1054R* (rs1800057) and *CHEK2*/*T470C* (rs17879961) polymorphisms and their putative association with the CAs, along with the evaluation of the health of 114 GSWs in Rio de Janeiro. The polymorphisms *RAD51/G135C*, *ATM/P1054R*, and *CHEK2*/*T470C* were selected because they were associated with many cancers, such as prostate, breast, head and neck cancer, and leukemias (Skasko et al. 2009; Schumacher et al. 2018; Zeng et al. 2018).

## Subjects and methods

### Population study

This is a cross-sectional study with 114 workers (60 men and 54 women) recruited at 11 gas stations in Rio de Janeiro and 115 age-matched controls (64 men and 51 women). A trained interviewer questioned the members of the study population regarding their age, sex, skin color (self-declaration), life-style (smoking habits, alcohol and illicit drug consumption, etc.) and about their offspring (Table 1). The control groups were recruited among administrative workers, cleaning workers, and nurses (not exposed to chemotherapy neither X-rays) of two hospitals, in a church (housewives and workers) and teaches. By the questionnaire, we did not detected high exposure to tobacco, alcohol consumption or industrialized food intake. Individuals showing alterations in the blood test were excluded from the control group. The minimum period of exposure for the GSW was 6 months. If the participant had undergone any kind of surgery, either was exposed to X-ray, or was infected by arboviruses in 3 months before the interview, man or woman was excluded from the study. No medication causing CA was reported by the participants. No test for virus was conducted in the blood of the subjects, nevertheless we asked for previous diseases. The subjects reported no hereditary condition although some have reported cases of cancer in the family. It was unclear if the cancer was hereditary. No significant difference was found in smoking cigarettes, alcohol consumption or industrialized food intake between the GSW group and the control´s.

**Table 1:** Demographics data of gas station workers

Data	Group 1	Group 2	Group 1 × Group 2 (*P* -value)	Total of workers	Controls	Total of workers × controls (*P *value**)**
Gender			**0.035**			0.235
Women	39 (42.4%)	15 (68.2%)		54 (47.4%)	64 (55.7%)	
Men	53 (57.6%)	7 (31.8%)		60 (52.6%)	51 (44.3%)	
Age (year)	38.9 (± 12.4)	38.8 (± 12.51)	0.900	38.84 (± 12.42)	36.43 (± 12.93)	0.101
Time of employment (year)	5.7. (± 6.0)	4.3 (± 3.8)	0.574			
Skin color			0.638			0.100
Black	25 (27.2%)	8 (36.4%)		33 (28.9%)	20 (18.2%)	
White	14 (15.7%)	3 (13.6%)		17 (14.9%)	48 (43.6%)	
Brown (Mulatto)	50 (54.3%)	10 (45.5%)		60 (52.6%)	41 (37.3%)	
Light brown (Native Indians)	2 (2.2%)	0 (0.0%)		2 (1.8%)	1 (0.9%)	
Yellow (Asiatic)	1 (1.1%)	1 (4.5%)		2 (1.8%)	0 (0.0%)	
Alcohol consumption						
No				18 (15.8%)	52 (45.2%)	1.000
Yes				75 (65.8%)	51 (44.3%)	
Stopped drinking				15 (13.2%)	5 (4.3%)	
Never drank				6 (5.3%)	7 (6.1%)	
First trimester of spontaneous abortion				7 (77.8%)	1 (11.1%)	**0.015**

*P * value < 0.05 was considered significant

Descriptive measures: *a* (± *b*), *a* = average and *b* = standard deviation

Peripheral blood samples were collected for complete hemogram, biochemistry and cytogenetic tests. The workers were divided into two groups (group one, ≤ 10 chromosomal abnormalities per 1,000 metaphases; and group two, > 10 chromosomal abnormalities per 1000 metaphases) and compared to clinical characteristics and genotyping results.

### Cytogenetic analyses

The cytogenetic analyses were performed for delimiting GSWs at risk as previously described and for allowing associations between the frequency of lymphocyte CAs, genotyping results, and clinical characteristics (Zhang et al. 2012; Verdorfer et al. 2001; Santiago et al. 2014). Blood samples, 2 mL of heparinized whole blood, were collected by venipuncture. Lymphocyte cultures were performed and chromosomes were prepared according to standard procedures after 48 h of cultivation (Liehr and Claussen 2002). FISH was done as previously reported using homemade wcp probes for chromosomes one, two, and four (Verdorfer et al. 2001; Santiago et al. 2014). One-hundred metaphases were analyzed per GSW and 200 metaphases in 11/115 controls.

### Genotyping

Genomic DNA from peripheral blood leukocytes was obtained by phenol–chloroform extraction and analyzed by polymerase chain reaction and restriction enzyme digestion (PCR–RFLP) assays for *RAD51*/*G135C* (rs1801320), *ATM*/*P1054R* (also known as 3161C > G, rs1800057), and *CHEK2*/*T470C* (rs17879961) polymorphisms according to previous publications (Skasko et al. 2009; Green and Sambrook 2012; Schumacher et al. 2018). The PCR reactions were carried out in the VeritiVR Thermal Cycler (Applied Biosystems) and were done using 50–200 ng of genomic DNA, 0.4 µM of each primer, 1 × PCR buffer, 250 µM of dNTPs, 1.5 mM of MgCl_2_, and 1–2.5 units of Taq polymerase in a 50 µL reaction volume. PCR products were digested with MvaI (*RAD51*/*G135C*, 60 °C for 1 h), AlwI (*ATM*/*P1054R*, 37 °C for 1 h), and PstI (*CHEK2*/*T430C*, 37 °C for 5 min) (New England Biolabs), and then separated by electrophoresis in 10% polyacrylamide, and the digested/separated products were further visualized by silver staining. Positive and negative controls were used in all reactions. Note that for *RAD51/G135C* polymorphism, the wild allele is represented by the letter "G" (Guanine) and the variant allele by "C" (Cytosine). While for ATM/P1054R polymorphism, the wild allele is represented by the letter "C" (Cytosine) and the variant allele by "G" (Guanine).

### Statistical analysis

The Hardy–Weinberg (HW) equilibrium was tested using the Chi-Square ($\chi^{2}$) statistic for the goodness-of-fit test for each polymorphism, and the differences in the allele and genotype frequencies between groups were analyzed using standard $\chi^{2}$ or Fisher’s exact tests. In the distributed variables, a nonparametric Mann–Whitney test or Goodness-of-fit test (multinomial distribution) was used for comparison of the distributed variables between groups using the IBM SPSS (version 2.0). The odds ratio (OR) was also calculated. For all statistical tests, *P* value < 0.05 was considered significant.

## Results

### Clinical and demographic data

The GSWs interviewed in this study routinely worked for six days a week, for eight hours or more per day, with 6.9 years of median time of employment. Regarding age, there were no significant differences between the workers (38.84 ± 12.42) and the control groups (36.43 ± 12.93) (*P* = 0.101). As for skin color (self-declaration), 52.6% (60/115) self-declared as brown (Mulatto), 14.9% (17/115) white, 28.9% (33/115) black, 1.8% (2/115) light brown (as Native Indians), and 1.8% (2/115) yellow (as Asiatic). A low prevalence of smoking (7%) and moderate consumption of alcohol beverage were identified. No statistical differences were found between alcohol consumption, illicit drug use (marijuana, cocaine, and ecstasy), smoking, and race between workers and controls (*P* = 1.000; *P* = 1.000; *P* = 0.293; *P* = 0.100, respectively; see Table 1). Despite no statistical difference for gender between workers and controls, a higher number of women were observed in group two (68.2%) compared to group one (42.4%) (*P* = 0.035).

Regarding the comparative analyses of blood tests, monocytes, eosinophils, basophils, hemoglobin (men), hematocrit (men), and gamma-gt were found to be significantly higher in the workers group, when compared with the controls (*P* = 0.001; *P* < 0.001; *P* < 0.001, *P* = 0.001, *P* = 0.003, and *P* < 0.001, respectively). On the other hand, platelets, erythrocytes (women), hemoglobin (women), and neutrophil levels (*P* = 0.001, *P* = 0.001, *P* = 0.003 and *P* = 0.001, respectively) were lower. It should be noted that only monocytes were associated with a high CA rate (*P* = 0.024, group one vs. group two), as showed in Table 2.

**Table 2:** Laboratory data of gas station workers

Data	Group 1	Group 2	Group 1 × Group 2 (*P* value)	Total of workers	Controls	Total of workers × controls (*P* value**)**
Platelets (10^9^/L)	254.16 (± 58.83)	255.52 (± 70.85)	0.969	254.43 (± 60.99)	278.29 (± 49.70)	**0.001**
Gamma-GT (U/L)	37.17 (± 39.40)	28.81 (± 12.61)	0.819	35.74 (± 35.86)	27.00 (± 37.30)	** < 0.001**
Direct bilirubin (mg/dL)	0.39 (± 0.17)	0.34 (± 0.15)	0.223	0.14 (± 0.05)	0.16 (± 0.07)	**0.046**
Leukocytes (/μL)	7318.1 (± 2102.3)	7226.2 (± 1291.2)	0.795	7300.22 (± 1966.01)	7778.61 (± 1980.38)	0.066
Neutrophils (%)	55.41 (± 9.80)	55.78 (± 8.36)	1.00	55.49(± 9.51)	59.46 (± 10.92)	**0.001**
Eosinophils (%)	3.10 (± 2.76)	2.73 (± 1.61)	0.951	3.03 (± 2.58)	2.14 (± 3.09)	** < 0.001**
Basophils (%)	0.42 (± 0.34)	0.33 (± 0.23)	0.577	0.40 (± 0.32)	0.27 (± 0.43)	** < 0.001**
Typical lymphocytes (%)	33.62 (± 8.92)	35.05 (± 7.89)	0.385	33.89 (± 8.72)	±32.44 (±9.79)	0.152
Monocytes (%)	7.40 (± 2.04)	6.24 (± 1.55)	**0.024**	7.18 (± 2.00)	5.42 (± 1.95)	** < 0.001**
Reticulocytes (%)	±1.13 (±0.38)	1.19 (±0.30)	0.209	1.15 (± 0.36)	1.27 (± 0.46)	0.080
Women						
Erythrocytes (million/μL)	4.48 (± 0.22)	4.31 (± 0.35)	0.148	4.44 (± 0.37)	4.62 (± 0.48)	0.056
Hemoglobin	13.06 (± 1.1)	12.54 (± 1.09)	0.117	12.92(± 1.11)	13.58 (± 1.88)	**0.021**
Hematocrit (%)	38.76 (± 2.98)	37.42 (± 3.02)	0.216	38.40(± 3.02)	40.18 (± 3.56)	**0.002**
Mean corpuscular volume (fl)	86.66 (± 5.21)	86.86 (± 3.73)	0.845	86.72 (± 4.82)	87.12 (± 5.13)	0.511
Men						
Erythrocytes (million/μL)	4.98 (± 0.37)	4.95 (± 0.11)	0.904	4.98 (± 0.35)	4.72 (± 0.45)	**0.001**
Hemoglobin	14.47 (± 1.13)	14.49 (± 0.71)	0.884	14.47 (± 1.08)	13.82 (± 2.08)	**0.003**
Hematocrit (%)	42.37 (± 2.86)	42.51 (± 1.55)	1.000	42.38 (± 2.72)	42.23 (± 3.71)	0.370
Mean corpuscular volume (fl)	84.90 (± 4.89)	84.90 (± 2.42)	0.645	85.42 (± 4.35)	88.07 (± 5.07)	**0.001**

*P * value < 0.05 was considered significant

Normal values: Platelets 150–400 10^9^/L; gamma-GT 8-71 U/L; direct bilirubin up to 0.3 mg/dL; leukocytes 4000–10,000/μL; neutrophils 40–75%; eosinophils 1–6%; basophils 0–1%; typical lymphocytes 20–45%; monocytes 2–10%, reticulocytes 0.5–2%; erythrocytes 4.5–6.5 million/μL; hemoglobin 13.5–18 g/dL, hematocrit 40–54%, mean corpuscular volume 76–96 fl. Descriptive measures: *a* (± *b*), *a* = average and *b* = standard deviation

### The CA data

The GSWs CA rate was 9.8 CAs/1000 metaphases, and a high frequency of CAs (> 10 CAs per 1000 metaphases) was found in 19.1% (22/114) of GSWs, whereas 80.9% (92/114) of workers showed no aberrations or less than ten CAs per 1000 metaphases, and no CAs were found among controls.

**Fig. 1:**
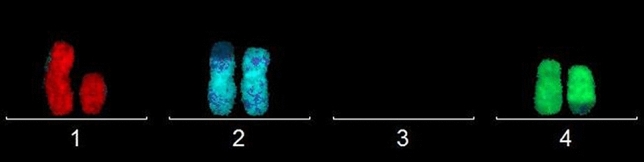
CAs found in analyses of one female worker. CAs—del(1),der(2),t(2;?), der(4),t(4;?). The homemade probes were conjugated with TexasRed to label chromosome 1 (red), Diethylaminocoumarin (DEAC) for chromosome 2 (lightblue), and fluorescein isothiocyanate (FITC) for chromosome 4 (green). Other chromosomes were counterstained with DAPI (dark blue)

Chromosome one with 38.4% (43/112) of CAs was the most affected, followed by chromosomes four (32.1%) and two (29.4%); however, no statistical difference was found between the chromosomes and CA distribution (*P* = 0.494). Among the total CAs, the translocations were most frequently found (38.4%), followed by monosomies (14.3%); deletions (13.4%); chromosomal fragments (13.4%); chromosomal breaks (11.6%); chromosome derivatives (5.3%); trisomies (1.8%), and inversion (1.8%). Figure 1 shows an example of CAs found in one female worker (CAs—del(1),der(2),t(2;?), der(4),t(4;?)).

### Genotyping

The *RAD51*/*G135C*, *ATM*/*P1054R*, and *CHEK2*/*T470C* polymorphisms were determined for GSWs and controls. The *RAD51*/*G135C* and *ATM*/*P1054R* polymorphisms did not show deviation from the HW equilibrium in the population analyzed (*P* = 0.322, *P* = 0.632, respectively), as shown in Table 3. However, the variant genotype (TC and CC) *CHEK2*/*T430C* was not found in GSWs or controls; thus, these results were not considered for statistical analysis. Neither *RAD51*/*G135C* nor *ATM*/*P1054R* polymorphisms were associated with gender or ethnicity. In a comparative population analysis, the frequencies of *ATM*/*P1054R* showed no statistical difference between total workers and controls (*P* = 0.930); however, by the Chi-Square test, the frequencies of *RAD51*/*G135C* were different (*P* = 0.011) (see Table 3), indicating higher frequency of the *RAD51*/*G135C* variant in the GSW population.

**Table 3:** Genotypic frequencies of *RAD51*/*G135C* and *ATM*/*P1054R* genotypes in 114 gas station workers and 115 controls

	*RAD51*/*G135C*			*ATM*/*P1054R*		
	GG	GC	CC	CC	GC	GG
Group 1	59 (64.1%)	29 (31.5%)	4 (4.3%)	70 (79.5%)	18 (20.5%)	0 (0.0%)
Group 2	9 (40.9%)	10 (45.5%)	3 (13.6%)	19 (86.4%)	2 (9.1%)	1 (4.5%)
Total of workers *N* (%)	68 (59.6%.)	38 (33.3%)	8 (7.0%)	89 (80.9%)	20 (18.2%)	1 (0.9%)
Controls *N* (%)	88 (72.2%)	24 −(21.1%)	2 (1.8%)	92 (82.1%)	20 (17.9%)	0 (0.0%)
Total	156 (68.4%)	63 (27.6%)	9 (3.9%)	181 (81.5%)	40 (18.0%)	1 (0.4%)
	*P* value			*P* value		
Hardy–Weinberg Equilibrium	0.322			0.632		
Total workers × controls	**0.011**			0.930		
Group 1 × Group 2	0.074			0.092		

Polymorphisms assessment for CA detection			
	*RAD51*/*G135C*	*ATM*/*P1054R*	*RAD51*/*G135C* + *ATM*/*P1054R*
Specificity (%)	87 (76–94)	79 (68–87)	82 (75–88)
Sensitivity (%)	28 (16–43)	14 (16–43)	24 (14–35)

*P * value < 0.05 was considered significant

Descriptive measures: *a* (± *b*), *a* = average and *b* = standard deviation. (*a*−*b*), confidence interval sample, 95%

There was a positive association for a number of CAs per GSW and variants of *RAD51*/*G135C* genotypes (*P* = 0.008, GG + GC × CC; *P* = 0.011; GG × CC; and *P* = 0.034, GC × GG), as shown in Table 4. Similar results were found for the distribution of the number of abnormal metaphases per workers (*P* = 0.005, GG × GC + CC; *P* = 0.004; GG × CC; and *P* = 0.028, GG × GC) (see Table 4).

**Table 4:** Associations between genotypic frequencies of *RAD51/G135C* in 114 GSW and biometrics (cytogenetic and demographic) data

	*RAD51*/*G135C*			*P* value GG × GC	*P* value GG × CC	*P *value GC + CC × GG
	GG	GC	CC			
Gender *N* (%)						
Men	35 (51.5%)	20 (52.6%)	6 (75.0%)	1.000	0.275	0.702
Ethnicity *N* (%)						
Women	33 (48.5%)	18 (47.4%)	2 (25.0%)			
Black	18 (26.5%)	12 (31.6%)	3 (37.5%)	0.555	0.164	0.395
Mulatto	40 (58.8%)	18 (47.4%)	3 (37.5%)			
White	9 (13.2%)	6 (15.8%)	1 (12.5%)			
Asiatic	0 (0.0%)	1 (2.6%)	1 (12.5%)			
Native Indians	1 (1.5%)	1 (1.5%)	0 (0.0%)			
Number of abnormal metaphases per subject	0.59 (± 1.4)	0.76 (± 1.00)	1.63 (± 1.6)	**0.028**	**0.004**	**0.005**
Number of chromosomal aberrations per subject	1.63 (± 1.92)	0.84 (± 1.94)	1.03 (± 1.33)	**0.034**	**0.011**	**0.008**

*P * value < 0.05 was considered significant

Descriptive measures: *a* (± *b*), *a* = average and *b* = standard deviation

Regarding the comparative analyses for types of chromosomal alterations and *RAD51*/*G135*C genotypes, we found a higher number of chromosome fragments (*P* = 0.004, GG × GC; *P* = 0.014; GG × GC + CC) and chromosome breaks (*P* = 0.013, GG × GC) between variant allele genotype groups (Table 5).

**Table 5:** Associations between genotypic frequencies of *RAD51/G135C* and types of chromosome alterations

	*RAD51*/*G135C*			*P *value GG × GC	*P* value GG × CC	*P *value GG × CC + GC
	GG	GC	CC			
Translocations						
0	58 (85.3%)	26 (68.4%)	6 (75.0%)	0.133	0.769	0.126
1	6 (8.8%)	7 (18.4%)	1 (12.5%)			
2	3 (4.4%)	4 (10.5%)	1 (12.5%)			
4	0 (0.0%)	1 (2.6%)	0 (0.0%)			
5	1 (1.5%)	0 (0%)	0 (0.0%)			
Chr. fragments						
0	64 (94.0%)	30 (78.9%)	8 (100.0%)	**0.004**	1.000	**0.014**
1	2 (2.9%)	8 (21.1%)	0 (0.0%)			
2	1 (1.5%)	0 (0%)	0 (0.0%)			
3	1 (1.5%)	0 (0%)	0 (0.0%)			
Chr. Breaks						
0	63 (94.0%)	35 (92.1%)	6 (75.0%)	0.787	**0.013**	0.245
1	3 (4.5%)	3 (7.9%)	0 (0.0%)			
2	0 (0.0%)	0 (0.0%)	2 (25.0%)			
3	1 (1.5%)	0 (0.0%)	0 (0.0%)			
Deletions						
0	62 (91.2%)	34 (91.9%)	6 (75.0%)	0.731	0.197	0.774
1	4 (5.9%)	3 (8.1%)	1 (12.5%)			
2	2 (2.9%)	0 (0.0%)	1 (12.5%)			
Chr. Derivatives						
0	65 (95.6%)	37 (97.4%)	8 (100.0%)	0.785	1.000	0.764
1	1 (1.5%)	1 (2.6%)	0 (0.0%)			
2	2 (2.9%)	0 (0.0%)	0 (0.0%)			
Inversions						
0	68 (100.0%)	36 (97.3%)	8 (100.0%)	0.352	1.000	0.398
1	0 (0.0%)	1 (2.7%)	0 (0.0%)			
Monosomies						
0	62 (91.2%)	36 (94.7%)	6 (75.0%)	1.000	0.248	1.000
1	4 (5.9%)	2 (5.3%)	1 (12.5%)			
2	1 (1.5%)	0 (0.0%)	1 (12.5%)			
5	1 (1.5%)	0 (0.0%)	0 (0.0%)			
Trisomies						
0	67 (98.5%)	37 (97.4%)	8 (100.0%)	1.000	1.000	1.000
1	1 (1.5%)	1 (2.6%)	0 (0.0%)			

*P * value < 0.05 was considered significant

Descriptive measures: *a* (± *b*), *a* = average and *b* = standard deviation

The frequencies of *ATM*/*P1054R* genotypes were compared between the workers and controls, and no significant difference was detected, indicating that the two populations were equivalent (Table 6). Only a weak positive association with chromosome breaks was detected, when compared between the genotypes with the variants CC × GG + CG (*P* = 0.054), as shown in Table 7. To assess the capacity of variant alleles *RAD51*/*G135C* and *ATM*/*P1054R* to detect the workers with CAs, the sensitivity and specificity were calculated. Note a considerable specificity for *RAD51*/*G135C* (87%) and *ATM*/*P1054R* (79%); however, lower sensitivity was found for both 28% and 14%, respectively. When the specificity was calculated for *RAD51*/*G135C* and *ATM*/*P1054R* together, the value found was 82% (see Table 3).

**Table 6:** Statistical analysis of *ATM*/*P1054R* genotypes

	*ATM/P1054R*			*P *value CC × GG + CG	*P *value CC × CG
	CC	CG	GG		
Gender *N* (%)					
Men	42 (47.2%)	14 (66.7%)	1 (100.0%)	0.097	0.146
Women	47 (52.8%)	7 (33.3%)	0 (0.0%)		
Ethnicity *N* (%)					
Black	25 (27.8%)	7 (33.3%)	0 (0.0%)	1.000	0.976
Brown	47 (52.2%)	11 (52.4%)	1 (100.0%)		
White	14 (15.6%)	3 (14.3%)	0 (0.0%)		
Asian	2 (2.2%)	0 (0.0%)	0 (0.0%)		
Native American	2 (2.2%)	0 (0.0%)	0 (0.0%)		
Number of abnormal metaphases per subject	0.74 (± 1.36)	0.62 (± 1.12)	1 (*N*)	0.924	0.913
Number of chromosomal aberrations per subject	1.01 (± 1.85)	0.71 (± 1.35)	2 (*N*)	0.517	0.965

Descriptive measures: *a* (± *b*), *a* = average and *b* = standard deviation

**Table 7:** Statistical analysis of *ATM*/*P1054R* genotypes and chromosome alterations

	*ATM*/*P1054R*			*P *value CC × CG	*P *value CC × GG + CG
	CC	CG	GG		
Translocations					
0	71 (78.9%)	16 (76.2%)	0 (0.0%)	0.771	0.781
1	10 (11.1%)	4 (19.0%)	0 (0.0%)		
2	7 (7.8%)	1 (4.8%)	0 (0.0%)		
4	1 (1.1%)	0 (0.0%)	0 (0.0%)		
5	1 (1.1%)	0 (0.0%)	0 (0.0%)		
Chr. Fragments					
0	78 (87.6%)	20 (95.2%)	0 (0.0%)	0.793	0.662
1	9 (10.1%)	1 (4.8%)	0 (0.0%)		
2	1 (1.1%)	0 (0%)	0 (0.0%)		
3	1 (1.1%)	0 (0%)	0 (0.0%)		
Chr. breaks					
0	84 (93.3%)	19 (90.5%)	0 (0.0%)	0.352	0.054
1	5 (5.6%)	1 (4.8%)	0 (0.0%)		
2	0 (0.0%)	1 (4.8%)	2 (100.0%)		
3	1 (1.1%)	0 (0.0%)	0 (0.0%)		
Deletions					
0	79 (87.8%)	20 (95.2%)	0 (0.0%)	0.275	0.279
1	9 (10.0%)	0 (0.0%)	0 (0.0%)		
2	2 (2.2%)	1 (4.8%)	0 (0.0%)		
Chr. derivatives					
0	87 (96.7%)	20 (95.2%)	0 (0.0%)	0.573	0.589
1	1 (1.1%)	1 (4.8%)	0 (0.0%)		
2	2 (2.2%)	0 (0.0%)	0 (0.0%)		
Inversions					
0	89 (98.9%)	21 (100.0%)	0 (0.0%)	1.000	1.000
1	1 (1.1%)	0 (2.7%)	0 (0.0%)		
Monosomies					
0	82 (91.1%)	19 (90.5%)	0 (0.0%)	0.799	0.808
1	5 (5.6%)	2 (9.5%)	0 (0.0%)		
2	2 (2.2%)	0 (0.0%)	0 (0.0%)		
5	1 (1.1%)	0 (0.0%)	0 (0.0%)		
Trisomies					
0	88 (97.8%)	21 (100.0%)	0 (0.0%)	1.000	1.000
1	2 (2.2%)	0 (0.0%)	0 (0.0%)		

Descriptive measures: *a* (± *b*), *a* = average and *b* = standard deviation

## Discussion

The association between two dysfunctional polymorphisms *RAD51*/*G135C* and *ATM*/*P1054R,* and CAs, as an early effect biomarker, was evaluated in this cross-sectional study. Numerous studies have associated exposure to BTEX with increased levels of CAs in circulating lymphocytes of exposed workers (Zhang et al. 2002, 2012; Santiago et al. 2014; Gonçalves et al. 2016). Increased levels of CAs have, in turn, been correlated with an increased risk of cancer, especially for hematologic malignancies, such as myelodysplastic syndrome (MDS) and acute myelogenous leukemia (AML)—(Smith 2010).

FISH using wcp probes was applied in our study to detect alterations caused by chronic exposure to BTEX in 21.87% (chromosomes 1, 2, and 4, together) of the human genome (Verdorfer et al. 2001). Similar results were previously described by our research group (Santiago et al. 2014) applying the same technique in GSW populations (rate: 9.3 CAs per 1000 metaphases), as well as results described by Verdorfer et al (2001) in populations exposed to nitroaromates (16.0 CAs per 1000 metaphases) and compared to controls (5.85 CAs per 1000 metaphases). No CA was detected in the control group, fact that draws attention when compared to the high CAs frequency found in GSW group. It is possible GSWs with higher rates of CAs have a higher risk of developing cancer in future than others with low rates of CAs.

In the present study, the frequencies of *RAD51*/*G135C* variant were higher in the GSW population when compared to controls, and the allele variant genotypes were associated with CAs per workers. In a meta-analysis study on the relationship between *RAD51*/*G135C* and cancer risk, Zhao and cowokers (2014) investigated 42 studies involving 19,142 cases and 20,363 controls (Zhao et al. 2014). They found a significantly increased risk for overall cancers and concluded that *RAD51*/*G135C* polymorphism is a candidate for susceptibility to cancer in general, especially for breast cancer. In another meta-analysis involving ten studies with, 656 patients and 3725 controls, the *RAD51*/*G135C* polymorphism was associated with increased MDS risk, while no association was observed for acute leukemia (He et al. 2014). In our study, chromosome fragments and chromosome breaks were positively associated with variant allele genotypes. There is evidence that in Rad51 deficient cells stop in the G2/M phase and accumulate chromosomal breaks prior to cell death or unregulated cell growth, justifying the association found (Sonoda et al. 1998; Mishra et al. 2018).

Regarding the *ATM* results, no differences were found in the proportion of carriers of the *ATM*/*P1054R* variant between workers and controls. However, this proportion was considerably higher among our workers (19.0%, 21 out of 110) compared to prostate cancer patients (9.5%, 25 out of 261) and controls (4.78%, 22 out of 460) described by Meyer and coworkers (2007). A weak positive association between chromosome breaks and the variant *ATM*/*P1054R* was detected in our workers, suggesting that more studies are necessary for a final conclusion.

In the case of the *CHEK2*/*T470C*, no variant alleles were found in our study, possibly due to the low frequency in our study population. *CHEK2*/*T470C* is associated with reduced DNA repair ability and increased cancer susceptibility, such as breast cancer, colorectal cancer and prostate cancer (Han et al. 2013; Kilpivaara et al. 2006; Dong et al. 2003). In the USA, the *CHEK2*/*T470C* variant has been reported in 1.2% of the population, while in Germany, the frequency was 2.2% in breast cancer cases and 0.6% in controls; and in Belarussian population, 5.7% in cases and 1.3% in controls (Bogdanova et al. 2005). It may be necessary to increase the number of workers to be analyzed to draw conclusions about the *CHEK2*/*T470C* polymorphism in the Brazilian GSWs.

The literature has also reported an influence of gene–gene interactions on cancer susceptibility. Several studies have shown that combinations of *RAD51* and *ATM* variants may increase the risk for cancer development (Hallajian et al. 2017). In our study, no increase in specificity or sensitivity was found for the *RAD51*/*G135C* and *ATM*/*P1054R* polymorphism combinations for detecting CAs. Perhaps for an effective GSWs genomic instability monitoring and an increase in the sensitivity and specificity in detecting CAs, it will be necessary to evaluate not only more polymorphisms related to the DNA repair, but also polymorphisms related to BTEX detoxification (Kanuoriya et al. 2015; Fang et al. 2017).

## Risk behavior and prevention of cancer

Hematological changes in classic blood tests were previously described (IARC 2018; Zhang et al. 2012; Silvestre et al. 2017). In the present study, a high rate of monocytes, eosinophils, basophils, and gamma-gt was found compared to controls, as previously described (Zhang et al. 2012; Mitri et al. 2015; Otero and Ornellas 2015). However, a lower rate of platelets and neutrophils was associated with the workers. Despite the higher rate of monocytes found in total of workers, a lower rate of monocytes was associated with a high number of CAs (group two), highlighting the importance of the simple classic blood test in monitoring their overall health. Recently, Getu et al (2020) studied GSW in Ethiopia. In disagreement to our study, they found that hematimetric values had a significant increment when compared with the control group. However, they considered that a larger sample size should be conducted to explore the impact of these chemicals on their population. So it will be useful to conduct a meta-analysis study to check points of agreement and disagreement in different world population.

We should also consider the high frequency of spontaneous abortions in the first trimester of pregnancy of total abortions reported by the female workers compared to female controls. This was also previously described by Silvestre and coworkers (2017) in a study with a lower number of female GSWs (Silvestre et al. 2017). Thus, the immediate absence of female workers to the gas station once pregnancy is confirmed is necessary to reduce the BTEX`s genotoxic and abortive effects. More maternal–child health studies are needed, since the female gender was associated with a higher number of CAs (group two). Women have shown faster benzene biotransformation than men, metabolizing 23–26% more benzene, and its known that benzene must be bio-transformed to exert its toxic effects. Thus, women may be at greater risk, and environmental/biological limit values established in studies of male subjects may be inadequate (Brown et al. 1998; Angelini et al. 2012; Moro et al. 2017; Santiago et al. 2017).

## Conclusion

Herein we describe a health survey and the consequent genome risks related to the chronic exposure to gasoline vapors as well as the possible ways to monitor such risks. CAs are standardized biomarkers used to identify not only the worker population at a higher risk of developing cancer, but also specific individuals who are susceptible to cancer development. The higher frequencies of the *RAD51*/*G135C* polymorphism in the GSW population and its association with higher CA frequency are a relevant result.

Increasing the knowledge of the DNA repair variations in maintaining the genomic stability and integrity in the GSWs is crucial for cancer prevention. As a result, a better understanding of inter-individual variations in susceptibility, with the identification of groups at higher risk, may provide a foundation for developing better prevention programs.

## Data Availability

The data used in the current study is available from the corresponding author on reasonable request.
